# Right ventricular papillary muscle crossing the moderator band mimicked infective endocarditis: the utility of multimodal imaging

**DOI:** 10.1093/omcr/omae026

**Published:** 2024-04-25

**Authors:** Masaki Kamiyama, Akinori Higaki, Ryo Miyabe, Haruhiko Higashi, Katsuji Inoue, Osamu Yamaguchi

**Affiliations:** Department of Cardiology, Pulmonology, Hypertension & Nephrology, Ehime University Graduate School of Medicine, Ehime, Japan; Department of Cardiology, Mimihara General Hospital, Osaka, Japan; Department of Cardiology, Pulmonology, Hypertension & Nephrology, Ehime University Graduate School of Medicine, Ehime, Japan; Department of Cardiology, Pulmonology, Hypertension & Nephrology, Ehime University Graduate School of Medicine, Ehime, Japan; Department of Cardiology, Pulmonology, Hypertension & Nephrology, Ehime University Graduate School of Medicine, Ehime, Japan; Department of Cardiology, Pulmonology, Hypertension & Nephrology, Ehime University Graduate School of Medicine, Ehime, Japan; Department of Cardiology, Pulmonology, Hypertension & Nephrology, Ehime University Graduate School of Medicine, Ehime, Japan

**Keywords:** cardiology and cardiovascular systems, infectious diseases and tropical medicine, radiology, nuclear medicine, and medical imaging

## Abstract

Right-sided infective endocarditis (RSIE) generally carries a positive prognosis; however, it can result in complications such as heart failure, underscoring the importance of prompt diagnosis. While echocardiography serves as the standard diagnostic tool, it may occasionally face challenges in distinguishing between normal structures and vegetations. In this report, we present the case of a 60-year-old man diagnosed with pyogenic vertebral osteomyelitis, alongside suspected coexisting RSIE. During both transthoracic and transesophageal echocardiography, a rod-like mobile structure was observed adjacent to the right ventricular moderator band. However, confirming its nature as an infective vegetation proved challenging. Despite the inconclusive diagnosis of IE by echocardiography, the positron emission tomography/computed tomography (PET/CT) scan and cardiac magnetic resonance imaging (MRI) played a pivotal role in distinguishing between normal structures and vegetations. Since IE could develop life-threatening events, the role of multimodal imaging is of paramount importance. This case serves as a compelling example of the diagnostic value through the integration of PET/CT and MRI in ruling out IE.

## INTRODUCTION

Right-sided infective endocarditis (RSIE) occurs less frequently and generally has a more favorable prognosis compared to left-sided infective endocarditis [1]. However, there are instances where RSIE can progress to heart failure, requiring surgical intervention [2]. Therefore, the prompt and accurate diagnosis of RSIE is crucial. Transthoracic echocardiography (TTE) is especially valuable for diagnosing RSIE, when it provides clear imaging of the right-sided cardiac structures. However, it may pose challenges when differentiating between normal right ventricular structures such as tricuspid complex, Chiari networks, Eustachian valves, and vegetations [3, 4]. In this report, we present a case of pyogenic vertebral osteomyelitis, raising a suspicion of RSIE. We encountered difficulties in distinguishing between normal structures and vegetations during echocardiographic examinations. However, the combined use of cardiac magnetic resonance imaging (MRI) and positron emission tomography/computed tomography (PET/CT) could reach to rule out RSIE and guiding us to the accurate diagnosis.

## CASE REPORT

The case involves a male patient in his 60s with a history of chronic hepatitis C, cirrhosis, and a prior splenectomy. He developed symptoms of fever, redness, and swelling around the right knee eight days after dental treatment. Initially, his primary physician opted for conservative management, prescribing oral clindamycin under the diagnosis of cellulitis. Despite some improvement in the redness, his inflammatory markers remained elevated, with a white blood cell count of 13 600/μl and a CRP level of 8.19 mg/dl, prompting his admission to our hospital fourteen days post-dental intervention.

Upon admission, the patient did not present symptoms of heart failure but complained of back pain. The electrocardiogram showed normal sinus rhythm and no ST-T change. He did not have any history of intravenous drug use, cardiac trauma, or congenital heart disease. Blood cultures on admission revealed the presence of *Streptococcus dysgalactiae* in 2 out of 2 sets. A chest and abdominal CT scan failed to identify the source of infection. As a result, the possibility of infective endocarditis as a source of the fever was suspected, leading to a referral to the cardiovascular department.

**Figure 1:**
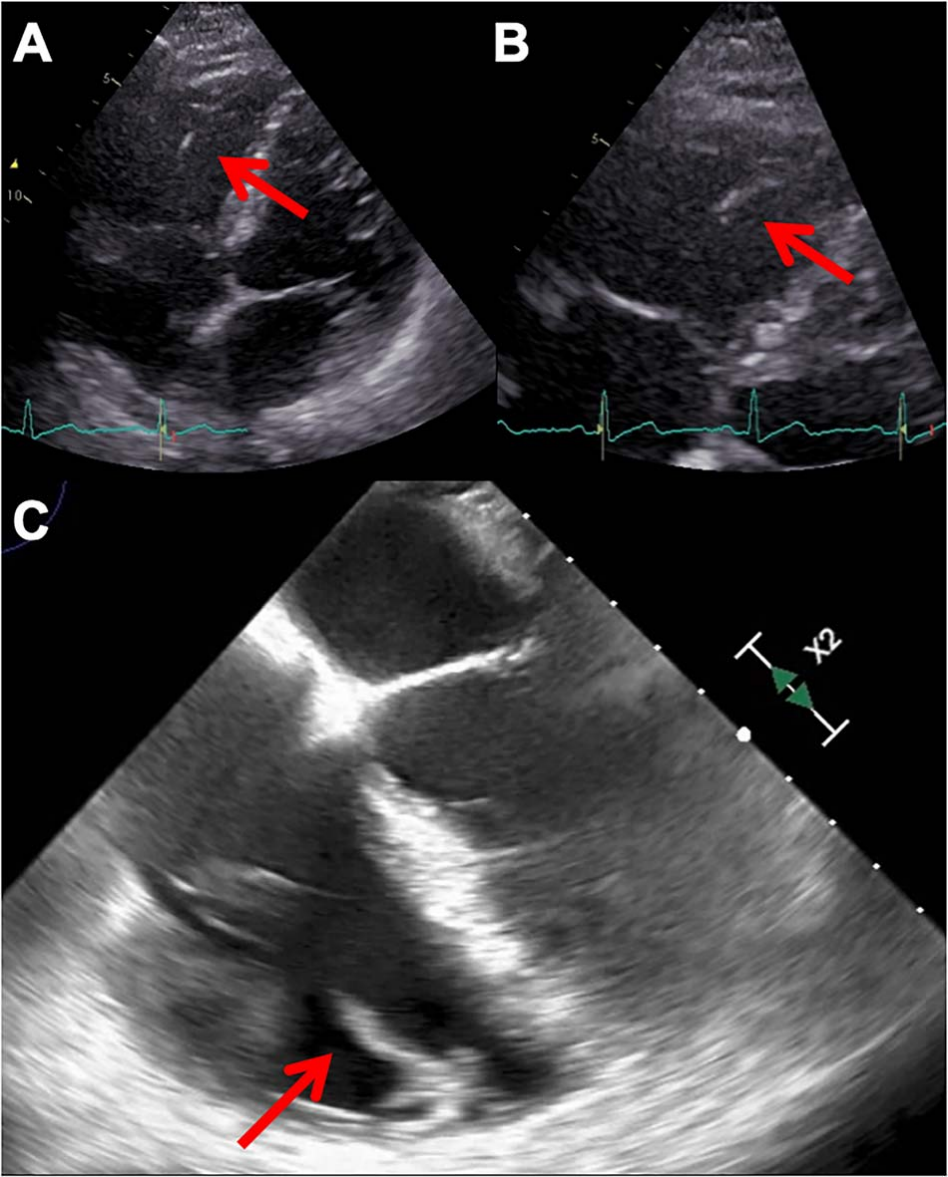
Echocardiographic visualization of suspected vegetation. Panels (**A**) and (**B**) display TTE images, with arrowheads denoting the suspected vegetation located in the apex of the right ventricle. Panels (**C**) provides TEE images, where arrowheads indicate the presence of a rod-like structure, raising the suspicion of vegetation.

**Figure 2:**
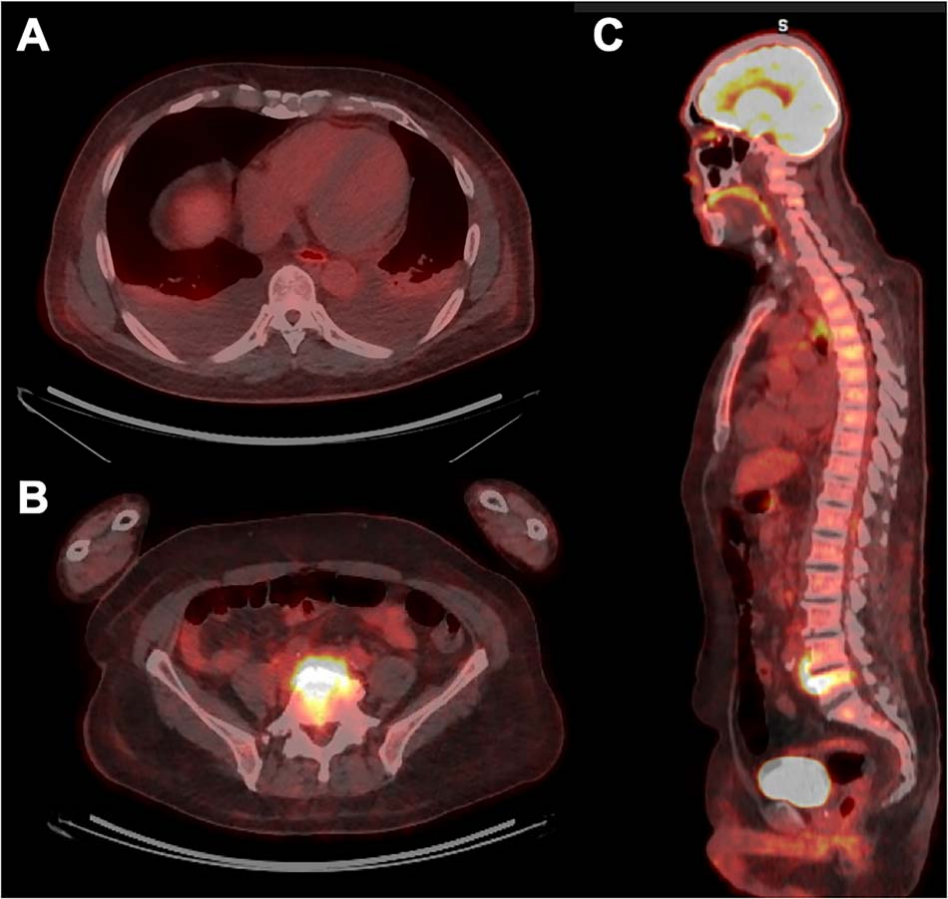
Images of FDG PET/CT. Absence of significant FDG uptake in the whole heart (panel **A**). Noticeable FDG uptake is observed in L4-5 vertebral bodies (panel **B** and **C**).

**Figure 3:**
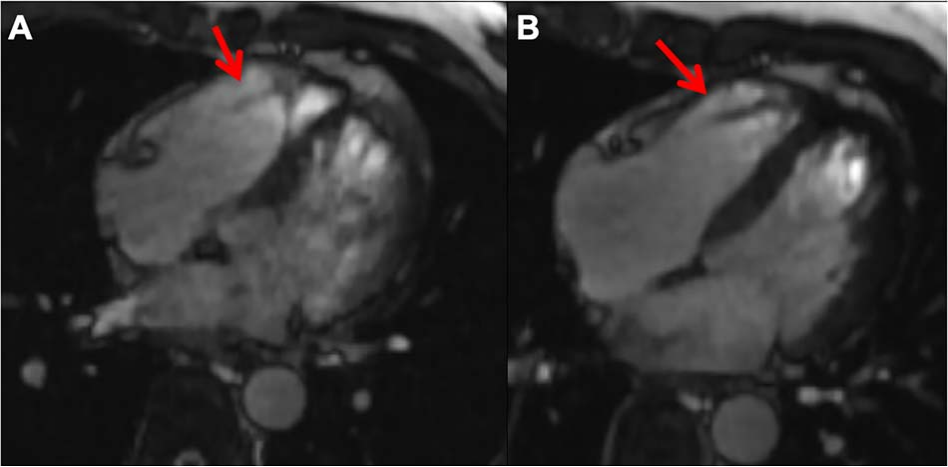
Cine cardiac MRI snapshots capturing the suspected vegetation site. In Panel (**A**), the image reveals the papillary muscle crossing the moderator band. Panel (**B**) highlights the extension of the papillary muscle towards the tricuspid valve leaflet, confirming it as normal structure. Arrowheads denoting the papillary muscle.

The TTE revealed preserved cardiac function without any shunt flow or significant valvular heart diseases. There were no mobile structures indicative of vegetation on the valves, except for a mobile and high-echoic structure in the right ventricular apex (Fig. 1A and B). Therefore, we considered the possibility of infective endocarditis and decided to perform transesophageal echocardiography (TEE). In the TEE, a mobile rod-like structure was observed adhering to the moderator band in the right ventricular apex (Fig. 1C). However, it was inconclusive to diagnose normal structure or vegetations. Therefore, to diagnose infectious endocarditis, we decided to perform fluorine-18-2-deoxy-D-glucose (FDG) PET/CT imaging to investigate systemic inflammatory sites throughout the body. Consequently, no uptake was detected within the cardiac chambers; however, there was notable FDG uptake observed in the L4-5 vertebral bodies (Fig. 2). Subsequently, the diagnosis of pyogenic vertebral osteomyelitis was confirmed, enabling to adequately deescalate the regimen of antibiotic treatment from meropenem to ampicillin. Nevertheless, we could not exclude the possibility of IE. To precisely assess the complex anatomical structures of the right sided heart, we performed a cardiac MRI (CMR) examination. The CMR revealed a rod-like structure in the right ventricle traversing the moderator band, and it connected with the tricuspid leaflet, confirming its identity as a papillary muscle. This structure appeared isointense with the myocardium and exhibited no high signal intensity on T2-weighted images (Fig. 3). Based on the above results, we determined the low probability of IE in this case. We could focus on the treatment for healing pyogenic spondylitis. The antimicrobial response was excellent, and blood cultures turned negative one week following hospitalization. On the 24th day, the patient was transferred to a secondary medical facility to receive ongoing antibiotic therapy and rehabilitation. The comprehensive clinical course is depicted in Table 1.

**Table 1:** Patient’s clinical timecourse

Timecourse	Clinical Events
14 days before admission	Dental treatment
7 days before admission	Fever and knee swelling
1 day before admission	Outpatient visit
Day 1	Hospital admission, plain CT, Positive blood culture
Day 2	Consultation with the cardiologist, initial TTE
Day 3	Transesophageal echocardiogram
Day 7	Follow-up TTE, Negative blood culture
Day 8	FDG PET/CT scan
Day 11	MRI of the spine
Day 12	Follow-up TTE
Day 23	Cardiac MRI
Day 24	Transfer to a backup hospital

CT, computed tomography; TTE, transthoracic echocardiogram; FDG PET/CT, fluorine-18-2-deoxy-D-glucose positron emission tomography/computed tomography; MRI, magnetic resonance imaging.

## DISCUSSION

Pyogenic vertebral osteomyelitis often presents a significant risk of complication, including IE. Previous reports have indicated that over 30% of patients diagnosed with pyogenic vertebral osteomyelitis have also developed IE [5]. In the present case, although we did not observe any vegetation during the initial TTE screening, we did identify a mobile rod-like structure attached to the moderator band. Given the precedent of mural endocarditis cases involving vegetation extending over the moderator band [6], we could not exclude the possibility of IE in our case.

Since IE is a life-threatening condition, and the accurate diagnosis by imaging modality is crucial for appropriate treatment. The use of FDG PET/CT enables the detection of increased glucose metabolism within organs and has proven valuable in diagnosing and monitoring inflammatory and infectious conditions. However, in native valve IE, the sensitivity of FDG PET/CT was reported as only 39% [7]. The reason for the relatively low sensitivity is its dependence on the size of vegetations, often leading to false negative finding when vegetations are small. Furthermore, low inflammatory activity has also been linked to false negative FDG PET/CT findings [8]. In our case, the blood culture returned negative a day before the PET/CT scan. Therefore, we did not exclude the possibility of IE and decided to combine with another imaging modality.

The CMR has been also considered to be useful in the diagnosis of infective endocarditis [9]. Cine MRI provides detailed images of valve issues and infections, especially when echocardiography faces challenges in understanding complex cardiac anatomy. Moreover, gadolinium contrast enhancement in the late phase enables the detection of myocardial damage resulting from IE [10]. In our case, the use of CMR allowed us to accurately identify the rod-like mobile structure as a papillary muscle connecting with the tricuspid valve.

FDG PET/CT and cardiac MRI alone may not necessarily have high sensitivity in detecting IE. However, it was considered possible to improve diagnostic performance by combining both methods. This case illustrates the efficacy of the combined use of CMR and PET/CT in ruling out right-sided IE in streptococcal sepsis.

## CONCLUSION

Right ventricular papillary muscle crossing the moderator band can mimic infective endocarditis in streptococcal infection. In such case, the integration of PET-CT and MRI constitutes a valuable tool for distinguishing vegetations from normal structures in right-sided heart.

## Supplementary Material

TEE1_omae026
